# Synergistic Effects of Combined Nurr1 Overexpression and Natural Inducers on the More Efficient Production of Dopaminergic Neuron-Like Cells From Stem Cells

**DOI:** 10.3389/fncel.2021.803272

**Published:** 2022-01-11

**Authors:** Reyhaneh Beiki, Mahsa Khaghani, Fariba Esmaeili, Fariba Dehghanian

**Affiliations:** Department of Plant and Animal Biology, Faculty of Biological Science and Technology, University of Isfahan, Isfahan, Iran

**Keywords:** stem cell differentiation, Nurr1 gene, tissue-specific microenvironment, soluble factors, dopaminergic neurons, Parkinson's disease

## Abstract

The development of dopaminergic (DA) neurons is a very complex process, and a combination of extrinsic and intrinsic factors involves their differentiation. Transcription factor, Nurr1 plays an essential role in the differentiation and maintenance of midbrain DA neurons. Nurr1-based therapies may restore DA function in Parkinson's disease (PD) by replacing damaged cells with differentiated cells derived from stem cells. Providing tissue-specific microenvironments such as brain extract can effectively induce dopaminergic gene expression in stem cells. The present study aimed to investigate the combined effects of Nurr1 gene overexpression and a neonatal rat brain extract (NRBE) induction on dopaminergic differentiation of P19 stem cells. In order to neural differentiation induction, stably Nurr1-transfected cells were treated with 100 μg/ml of NRBE. The differentiation potential of the cells was then evaluated during a period of 1–3 weeks via various methods. The initial evaluation of the cells by direct observation under a light microscope and cresyl violet specific staining, confirmed neuron-like morphology in the differentiated cells. In addition, different molecular and cellular techniques, including real-time PCR, immunofluorescence, and flow cytometry, demonstrated that the treated cells expressed pan-neuronal and dopaminergic markers. In all experimental groups, neuronal phenotype with dopaminergic neuron-like cells characteristics mainly appeared in the second week of the differentiation protocol. Overall, the results of the present study revealed for the first time the synergistic effects of Nurr1 gene overexpression and possible soluble factors that existed in NRBE on the differentiation of P19 stem cells into dopaminergic neuron-like cells.

## Introduction

*In vitro* production of midbrain dopamine neurons from stem cells is a promising approach for managing and treating Parkinson's disease (PD). The study of dopaminergic (DA) neuron development is one of the areas of interest in order to generate these neurons from stem cell sources. Although many pieces of research focused on *in vivo* and *in vitro* studies of these neurons for different purposes, still further investigations are needed to realize the factors that control DA cell differentiation and patterning. The development of DA cells is a very complex process, and a combination of extrinsic (sonic hedgehog, fibroblast growth factor 8, transforming growth factor b and Wnts) and intrinsic (En1/2, Lmx1a/b, Foxa1/2, Pitx3, and Nurr1) factors involves in their differentiation (Yuan et al., 2015). Previous studies have demonstrated that orphan nuclear receptor Nurr1 (nuclear receptor-related 1 protein, also known as NR4A2; nuclear receptor 4A2), as a transcriptional factor, plays an essential role in the differentiation and maintenance of midbrain DA neurons. Nurr1 activates tyrosine hydroxylase (TH) gene expression via direct binding to its promoter (Kim, 2011), and Nurr1 gene defect is associated with Parkinson's disease (Dong et al., 2016). DA neurogenesis failed in Nurr1 mutant mice and resulted in their death immediately after birth (Hermanson et al., 2003). Although Nurr1 heterozygous mice are healthy, dopamine levels reduced significantly in their ventral midbrain and striatum (Zetterström et al., 1997). Exogenous Nurr1 inserted in neural stem cells (NSCs) induced TH expression in a concentration-dependent manner (Kim et al., 2007). On the other hand, factors such as parathyroid hormone and corticotropin-releasing factor can induce Nurr1 expression in bone cells and a pituitary cell line, respectively (Murphy and Conneely, 1997; Tetradis et al., 2001). Nurr1-based therapies through replacing damaged cells by *in vitro* differentiated cells might restore DA functions in PD (Bartus et al., 2013). It has been previously reported that injection of Nurr1-overexpressing cells in combination with glial-derived neurotrophic factor (GDNF) or neurturin into the midbrain might protect DA neurons against toxicity of α-synuclein (Decressac et al., 2012; Bartus et al., 2013).

In order to produce highly specialized cells, it will be beneficial to create an environment similar to natural *in vivo* conditions. Numerous reports have shown that tissue-specific microenvironment can induce stem cell differentiation into appropriate cell types with the desired characteristics (Rivera et al., 2006; Bentz et al., 2010; Han et al., 2014; Narayanan et al., 2014; Momendoust et al., 2019). Recently, several studies have indicated that the use of a neonatal rat brain extract (NRBE) alone or in combination with a three-dimensional culture system (Azizi et al., 2018) or a neuroprotective drug, deprenyl (Momendoust et al., 2019), could effectively induce DA phenotype with the ability to express TH gene in stem cells.

Given the importance of Nurr1 as a key gene for DA neuron development, the present study was designed to investigate the effects of Nurr1 overexpression on dopaminergic differentiation of P19 embryonal carcinoma (EC) stem cells. In addition, stable Nurr1-transfected cells were exposed to NRBE to evaluate the synergistic effects of combined gene overexpression technology with a natural tissue-specific medium on the more efficient production of DA cells.

## Materials and Methods

### Culture and Stable Transfection of P19 Cells

A teratocarcinoma stem cell line, P19 (Cell Bank, Pasteur Institute of Iran, Tehran, Iran), was grown in α-MEM (alpha minimum essential medium, Gibco-BRL, Carlsbad, CA, 11900073) containing 10% FBS (fetal bovine serum, Gibco, 10270-106), 50 μg/ml penicillin (Sigma, P3032) and 50 μg/ml streptomycin (Sigma, S1277). A CaPO_4_ (calcium phosphate) co-precipitation method was used for transfection of the cells as previously described (Chen and Okayama, 1987). Briefly, the exponentially growing cells were trypsinized and seeded (1.5 × 10^6^) onto a 60-mm culture dish 24 h before transfection. A solution of CaPO_4_-DNA (10–20 μg total plasmid DNA; pCMV3-C-NR4A2-GFPSpark, Sino Biological, HG14938-ACG) was added dropwise onto the cells at 60–70% confluency. The cells were then incubated at 37°C in 5% CO_2_, and the growth medium was replaced by fresh α-MEM after 7–9 h. For stable transfection, a medium containing 200 μg/ml hygromycin B (Sigma, H0654) was applied, and the transfected cells were selected. Eight days after transfection, live cells were examined and photographed using a fluorescence microscope to detect GFP expression. P19-GFP^+^ cells were also generated by pCMV3-GFPSpark plasmid (empty) and considered as transfection control.

### Preparation of Neonatal Rat Brain Extract

Wistar rat strain was obtained from Isfahan University of Medical Sciences (Isfahan, Iran) and kept under standard housing conditions with a regular dark/light cycle. The animal procedures in the present study were performed according to the rules and regulations set by the Bioethics Committee of the University of Isfahan (Code: IR.UI.REC.1397.125), based on the National Specific Ethical Guidelines for Biomedical Research issued by the Ministry of Health and Medicinal Education (MOHME) of Iran in 2005. The rats were mated overnight, and the day neonates were born was considered as P0. One-week-old neonates sacrificed, their whole brains removed from the skull, and brain extracts prepared as described previously (Momendoust et al., 2019). Briefly, the whole brain was homogenized in a protease inhibitor (PMSF, Roche, 10 837 091 001) solution and then centrifuged at 3,000 rpm (10 min) and 12,000 rpm (20 min). All collected NRBE was pooled and total protein concentrations were assayed using the Bradford method. Finally, the extract was stored at −70°C until use.

### Neural Differentiation Induction Protocol

To neural differentiation of P19 cells, the cells were cultured in suspension for 3 days to induce embryoid bodies (EBs) production. The resulted EBs were transferred to gelatin-coated tissue culture dishes and allowed 1 day to attach. Four experimental groups were designed as follows: (1) E (empty): EBs transfected with pCMV3-GFPSpark vector (α-MEM + 5% FBS + empty plasmid); (2) EEx: EBs transfected with pCMV3-C-GFPSpark vector and treated by NRBE (α-MEM + 5% FBS + empty plasmid + 100 μg/ml NRBE); (3) N: EBs transfected with pCMV3-C-NR4A2-GFPSpark vector (α-MEM + 5% FBS + NR4A2 plasmid); (4) NEx: EBs transfected with pCMV3-C-NR4A2-GFPSpark vector and treated by NRBE (α-MEM + 5% FBS + NR4A2 plasmid + 100 μg/ml NRBE). The differentiation potential of the cells was evaluated during a period of 1–3 weeks.

### Specific Staining of the Differentiated Cells

The initial identification of the differentiated cells with neuronal phenotype was evaluated by: (1) direct observation of cell morphology under a light microscope; and (2) cresyl violet specific staining (Fraichard et al., 1995). To confirm the existence of Nissl bodies in the cytoplasm, the cells were fixed in 70% ethanol for 10 min at room temperature, dehydrated in 95% ethanol/5% acetic acid for 20 min at −20°C, and then exposed to cresyl violet solution (0.25% cresyl violet, 0.8% glacial acetic acid, 0.6 mM sodium acetate). Cells were washed in PBS (phosphate buffer saline) and then mounted for further examination.

### Real-Time PCR

To quantify the relative mRNA expression level of the interested genes, total RNA was extracted from all the samples, including untreated and treated groups. Complementary DNA synthesized (Parstous, Easy^TM^ cDNA Synthesis Kit, A101161), and real-time PCR assay was performed by StepOnePlus™ Real Time PCR System using specific primers (Table 1) and SYBR Premix Ex Taq (Takara, RR081Q). To analyze PCR reaction efficiency, standard curves were used for each gene, and a melt curve analysis was performed at the end of each reaction. The gene expression profile was quantified by the convenient 2^ΔΔCt^ method. Triplicate samples of cells were collected at each time point, and real-time PCR was performed on the corresponding synthesized cDNA. The Ct values provided by the Q-PCR instrument were easily imported into a spreadsheet program such as Microsoft Excel. The data were analyzed using amount of target = 2^−ΔΔCt^, [^ΔΔ^ Ct = (Ct, Target - Ct, housekeeping)_treatment_ - (Ct, Target - Ct, housekeeping)_control_]. Expression levels of the genes including stem cell markers, sex-determining region Y (SRY) box 2 (Sox-2), POU class 5 homeobox 1 (Oct3/4) and Nanog homeobox (Nanog) (Khoo et al., 2013); a neuroepithelial marker, nestin (NES); neural specific markers, synaptophysin (SYN), brain-derived neurotrophic factor (BDNF), nerve growth factor (NGF) and cAMP responsive element binding protein 1 (CREB1); and dopaminergic specific markers, tyrosine hydroxylase (TH), Nurr1 (NUR), LIM Homeobox Transcription Factor 1 Alpha (Lmx1a), LIM Homeobox Transcription Factor 1 Beta (Lmx1b), and forkhead Box A2 (Foxa2) and Girk2 (also known as KCNJ6, potassium voltage-gated channel subfamily J member 6), and onecut (ONC) were quantified and normalized to individual internal control, glyceraldehyde-3-phosphate dehydrogenase (GAPDH) as a housekeeping gene. The profile was obtained by plotting relative gene expression levels compared to the undifferentiated P19 cells.

**Table 1:** Real time PCR primer sequences.

**Gene**	**Accession number**	**Primers**	**Product size**
Sox2	NM_011443 XM_985079	F:TGAACCAGCGCATGGACAGCTA	221
		R:AGCCGTTCATGTAGGTCTGCGA	
Oct3/4	NM_013633	F:CTTCACCACACTCTACTC	156
		R:CCAGGTTCTCTTGTCTAC	
Nanog	XM_011241471	F:TGGGAACGCCTCATCAATGCCT	240
		R:CGCATCTTCTGCTTCCTGGCAA	
Nestin	NM_016701	F:TCAACCCTCACCACTCTATTTT	143
		R:GCTGTTTTCTACTTTTACCTCTGTG	
Synaptophysin	NM_009305	F:TGGCCACAGCAGTGTTCGCT	217
		R:ACCCAGAGCACCAGGTTCAGGA	
CREB	NM_009952.2	F:AGAAGCAGCACGGAAGAGAG	250
		R:CTTTCTGGTTGTGGCCAAGC	
Tyrosine hydroxylase	NM_009377	F:TGCAGCCCTACCAAGATCAAAC	103
		R:CGCTGGATACGAGAGGCATAGTT	
Nurr1	XM_011239033.2	F:CAGCTCCGATTTCTTAACTCCAG	159
		R:AGGGGCATTTGGTACAAGCAA	
Onecut	NM_008262	F:GGCAACGTGAGCGGTAGTTT	159
		R:TTGCTGGGAGTTGTGAATGCT	
BDNF	BC034862	F:GAAAGTCCCGGTATCCAAAG	181
		R:CCAGCCAATTCTCTTTTTGC	
NGF	BC011123.1	F:TTTCAACAGGACTCACCGGA	224
		R:TCTCAACAGGATTGGAGGCT	
β-actin	NM_007393	F:CCAACCGTGAAAAGATGACC	124
		R:GAGTCCATCACAATGCCAGT	
GAPDH		F:CGGCCGCATCTTCTTGTG	94
		R:TGACCAGGCGCCCAATAC	

### Immunofluorescence

Evaluation of the specific protein profiles of the differentiated cells carried out by immunofluorescence. The cells of all the samples were fixed in 4% paraformaldehyde and rinsed with PBS. Then, they permeabilized by 0.3% Triton X-100 and incubated with blocking buffer (10% normal goat serum; NGS, Sigma, G9023). The cells subsequently incubated with primary antibodies, including rabbit polyclonal nestin (Sigma, N5413), mouse anti-synaptophysin monoclonal (Abcam, ab8049), mouse anti-β-III tubulin monoclonal (Abcam, ab7751), rabbit anti-tyrosine hydroxylase–neuronal marker (Abcam, ab6211), rabbit polyclonal antibody to GFP (Abcam, Cambridge, USA, ab290), and mouse monoclonal anti-Nurr1 antibody (Abcam, ab41917), each at 1:1,000 dilutions. FITC-conjugated anti-mouse IgG (Sigma, F9137) and Cy5.29-conjugated anti-rabbit IgG (Abcam, ab6564) each at 1:1,000 dilutions applied as secondary antibodies. DAPI (4′,6-Diamidino-2-phenylindole, Sigma, D9542) utilized to counterstain the nuclei.

### Flow Cytometry

The cells were washed with PBS and gently dissociated into a single-cell suspension using 0.25% trypsin/EDTA (Sigma, T4799). Fixation buffer (4% paraformaldehyde in PBS) was added to 100 μL cell suspension, and then the cells were permeabilized by Triton X-100 and incubated with blocking buffer (10% NGS in PBS). The cells were exposed to above mentioned primary and secondary antibodies. Un-transfected cells were used to adjust the detector settings. Fluorescent intensity was determined on FACS (BD FACSCalibur), with 10,000 events captured per sample. The interested cell population, excluding debris and dead cells, was determined by forward and side scatters gating. The acquisition and analysis of the FACS data were performed with FlowJo software.

### Statistical Analysis

Statistical differences were evaluated between two groups by Student's *t*-test and multiple groups by one-way ANOVA (analysis of variance) and LSD (least significant difference) test. The experiments were performed in triplicate, and the data presented as mean ± SD (standard deviation) and *p* < 0.05 considered significant.

## Results

### Morphological Studies of the Differentiated Cells

**Figure 1:**
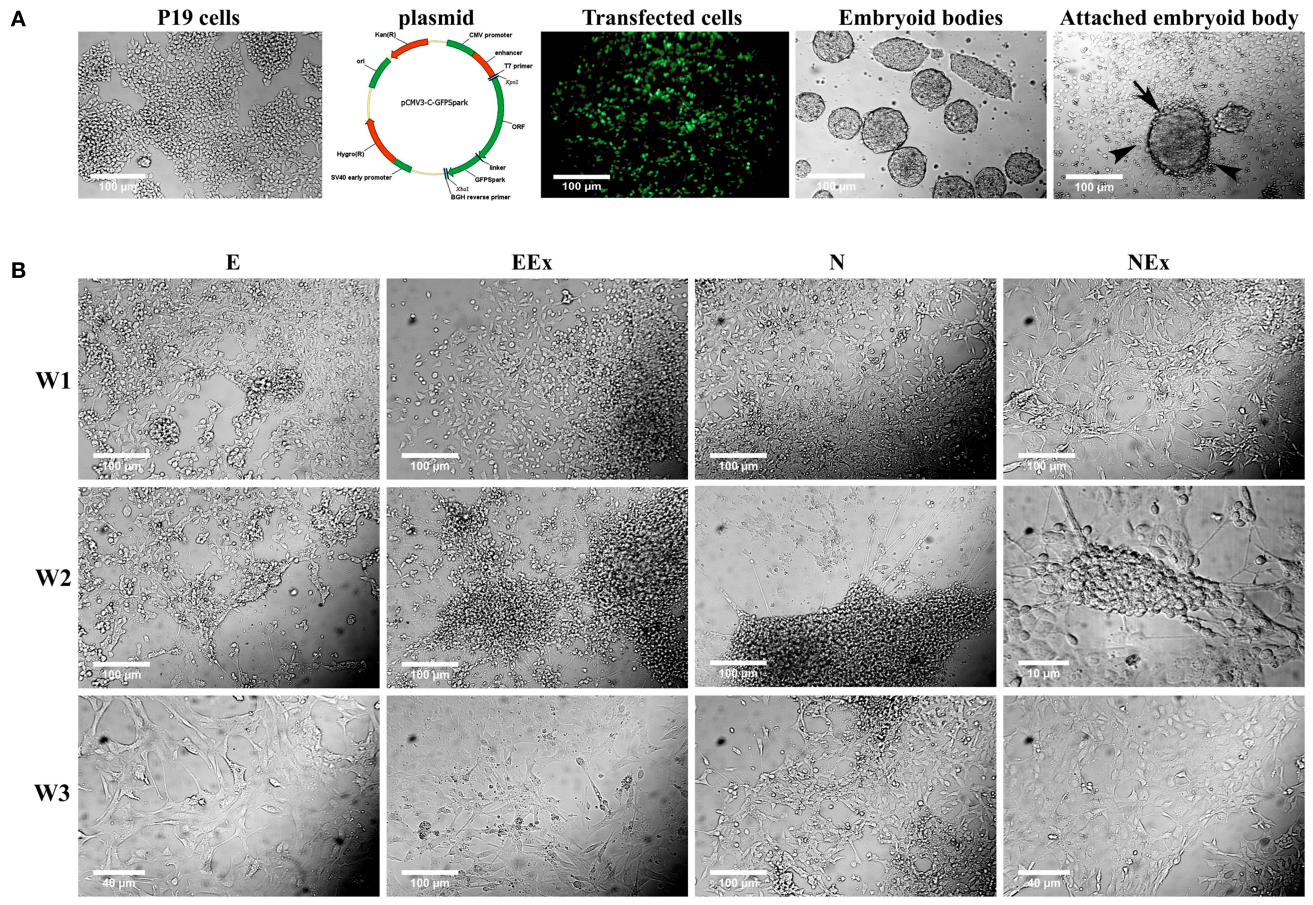
Initial morphological evaluation of neuronal phenotype. **(A)** P19 EC cells were stably transfected, and GFP+ EBs transferred on gelatin-coated culture dishes. Attached EBs (arrow) and the cells getting out radially (arrowheads) from them were then exposed to four various conditions. **(B)** Visual examinations of the photomicrographs confirmed that in all experimental groups, the neuronal phenotype was mainly appeared in the second week of the differentiation protocol, while it changed to non-neuronal appearance in the third week. The differentiated neuron-like cells have small cell bodies and numerous processes which form a cellular network with each other. E (empty), EBs transfected with pCMV3-GFPSpark vector; EEx (empty/extract), EBs transfected with pCMV3-GFPSpark vector and treated by extract; N (Nurr), EBs transfected with pCMV3-NR4A2-GFPSpark vector; NEx (Nurr/extract), EBs transfected with pCMV3-NR4A2-GFPSpark vector and treated by extract; W1, W2, W3, week 1, 2, 3, respectively.

To induce neural differentiation of P19 cells, they were stably transfected with a vector containing Nurr1 gene and GFP reporter gene under the control of cytomegalovirus (CMV) promoter. The expression of GFP in the transfected cells was confirmed by using a fluorescence microscope (Figure 1A). The embryoid bodies resulted from the suspension culture of GFP+ cells were transferred on gelatin-coated culture dishes and exposed to four various conditions as mentioned above. Initially, the morphological changes of the differentiated cells were evaluated using a light microscope over 1–3 weeks (Figure 1B). The cells with small bodies and numerous processes which formed a cellular network with each other were considered neuron-like cells. Although cell counting was not carried out here, visual examinations of the images showed that in all experimental groups, neuronal phenotype mainly appeared in the second week of the differentiation protocol. By the third week of the differentiation, the morphology of most cells changed to a non-neuronal appearance with large polyhedral cell bodies and a few short processes. There were very few cells with neuronal phenotype in the E group that showed spontaneous cell differentiation.

**Figure 2:**
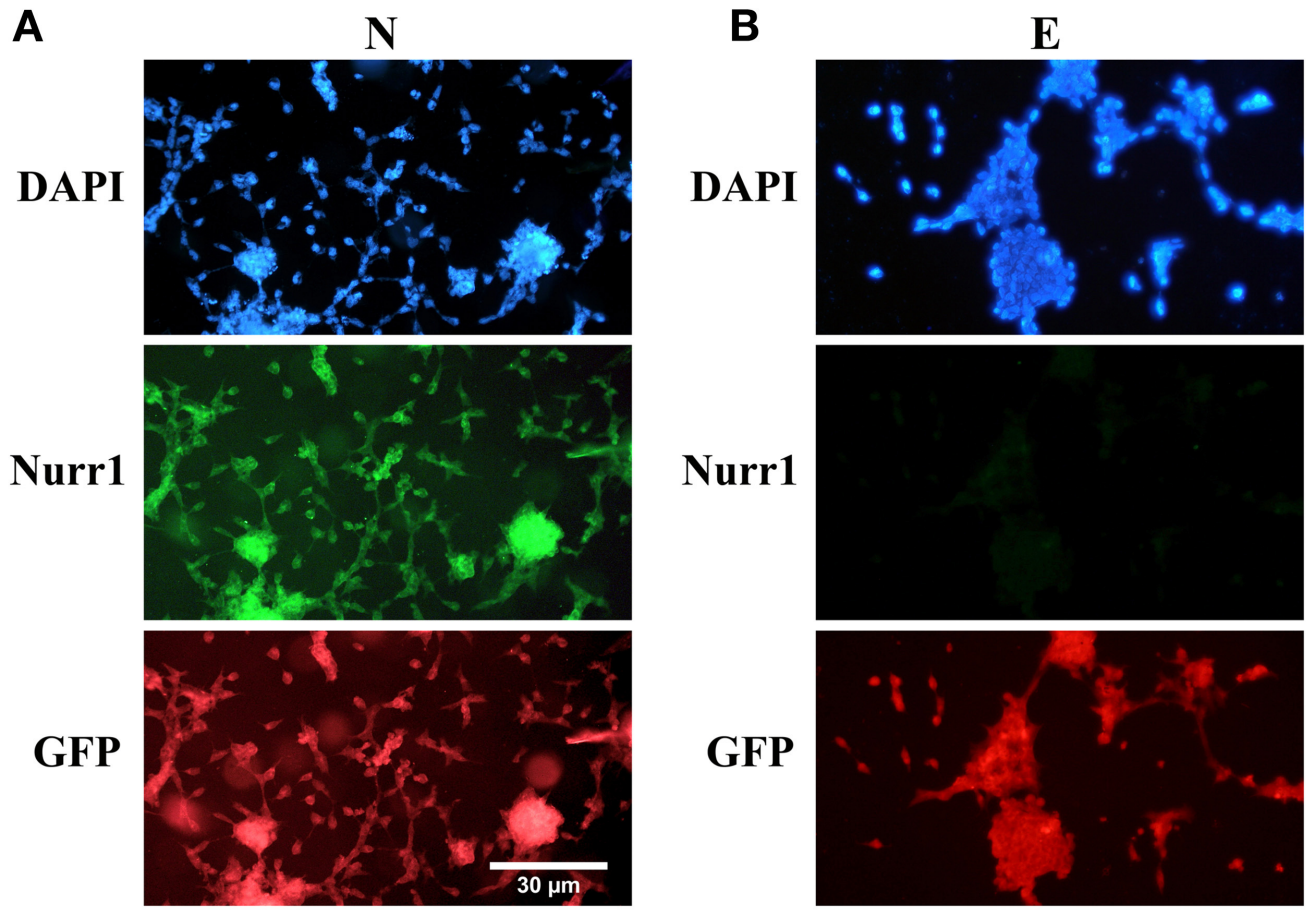
Double immunofluorescence staining with antibodies against Nurr1 and GFP. **(A)** The results confirmed co-expression of Nurr1 and GFP in the cells of N group (transfected by pCMV3-NR4A2-GFPSpark vector), **(B)** while the cells of E group (pCMV3-GFPSpark vector-transfected cells) showed no immunoreactivity to Nurr1 antibody.

Furthermore, double immunofluorescence staining of the paraformaldehyde-fixed cells with antibodies against Nurr1 and GFP confirmed co-expression of Nurr1 and GFP in the cells transfected by pCMV3-NR4A2-GFPSpark vector (Figure 2A), while pCMV3-GFPSpark vector-transfected cells showed no immunoreactivity to Nurr1 antibody (Figure 2B).

**Figure 3:**
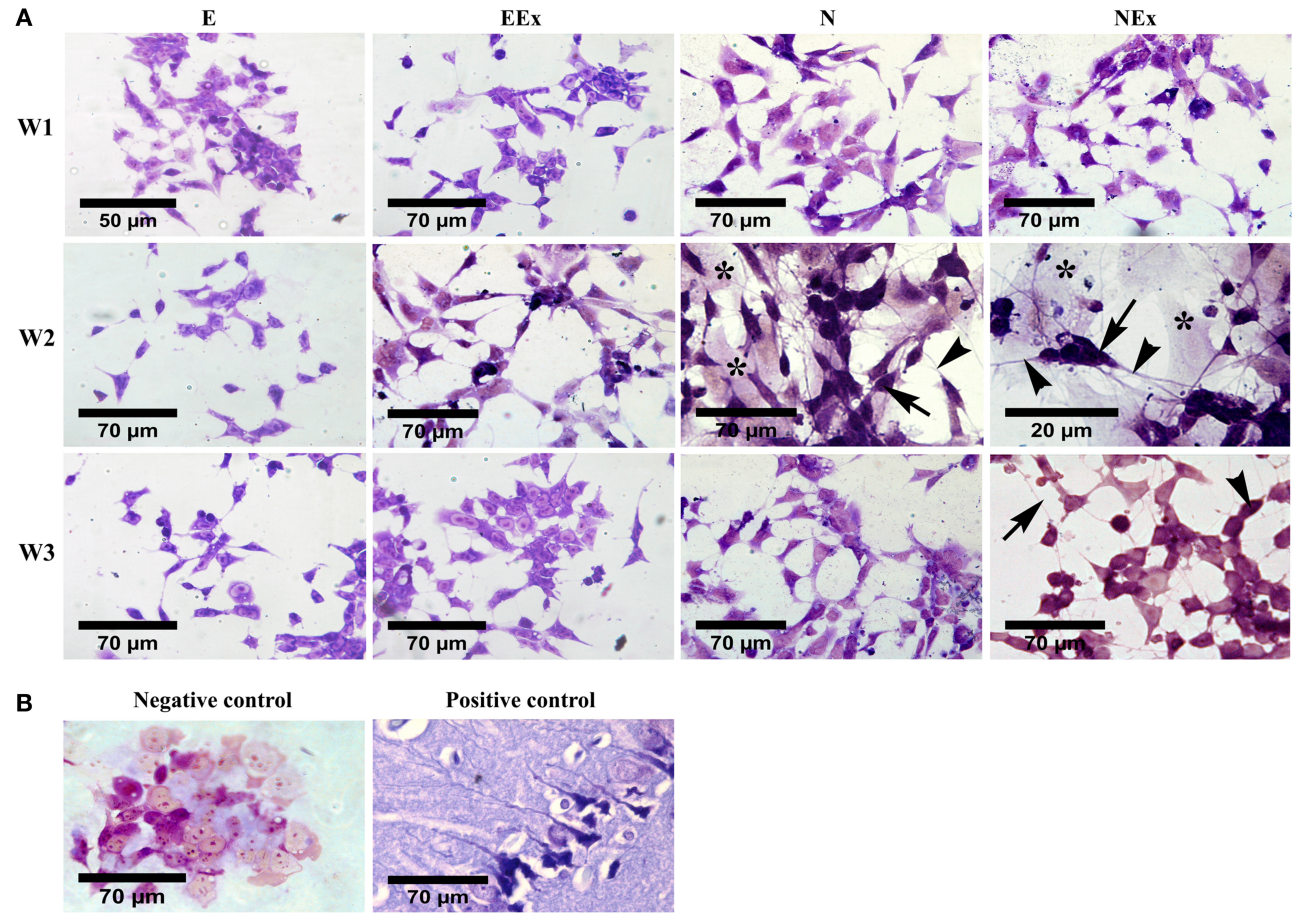
Evaluation of neural phenotype in the differentiated cells by cresyl violet specific staining. **(A)** Over a period of 1–3 weeks, the cells in all the experimental groups stained, and purple cells with a small body (arrows) and fine processes (arrowheads) were considered as neuron-like cells. Examination of the cells using a bright-field microscope indicated that the density of the cells with neural phenotype was higher in the second week of the differentiation protocol, while, in the third week, they showed a non-neuronal appearance. The purple cells were interconnected individually or as aggregates via their numerous fine processes to form neuron-like networks on top of a bed of pale cells with a non-neural appearance (asterisks). **(B)** P19 untreated cells and rat brain tissue were also stained with cresyl violet and presented as negative and positive controls, respectively. E (empty), EBs transfected with pCMV3-GFPSpark vector; EEx (empty/extract), EBs transfected with pCMV3-GFPSpark vector and treated by extract; N (Nurr), EBs transfected with pCMV3-NR4A2-GFPSpark vector; NEx (Nurr/extract), EBs transfected with pCMV3-NR4A2-GFPSpark vector and treated by extract; W1, W2, W3, week 1, 2, 3, respectively.

Morphological evaluation of the differentiated cells was further carried out by cresyl violet dye which specifically stains Nissl substances in the cytoplasm of neurons (Figure 3). The results confirmed that most cells in all experimental groups showed a positive response to this dye. The purple neuron-like cells, with their small cell body and numerous processes, formed a cellular network on top of pale-stained large flat cells with a non-neuronal appearance (Figure 3A). Interestingly, the clusters of neuron-like cells were interconnected by their fine processes. A few neuron-like cells were observed in the E group, possibly due to spontaneous differentiation. Representative photomicrographs were also shown as negative (P19 untreated cells) and positive (rat brain tissue) controls (Figure 3B).

### Evaluation of Gene Expression by Real-Time PCR

**Figure 4:**
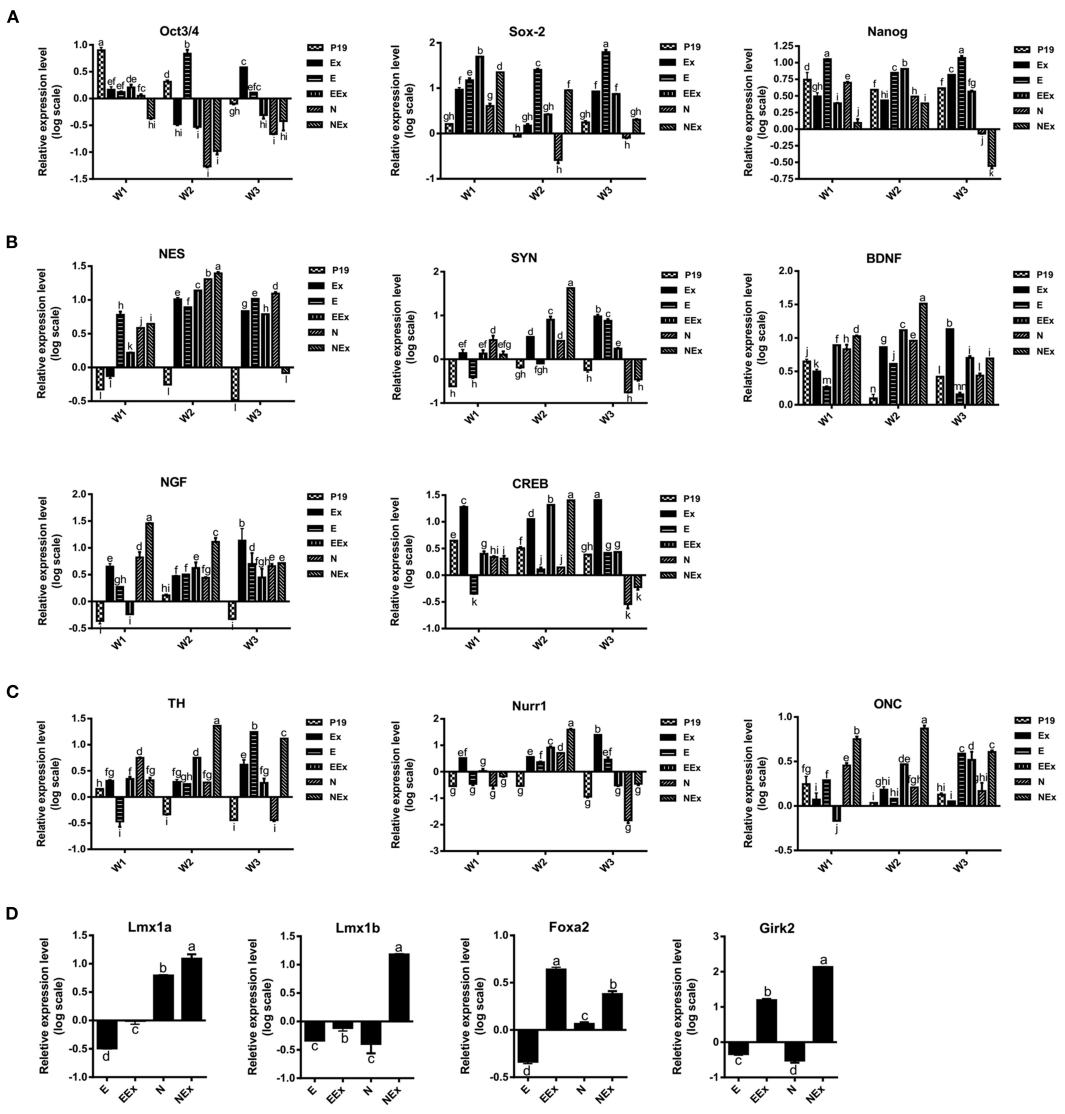
Evaluation of gene expression profile in the experimental groups by quantitative Q-PCR. **(A)** The expression of stem cell markers tend to decrease in treatment groups. Sox-2 expression was lowest in the N group, and the lowest level of Oct3/4 gene expression was in N, NEx, EEx, and Ex groups during the second week of differentiation. Nanog gene expression was at its lowest level in the NEx group in the third week. **(B)** The level of neuroepithelial (nestin) and neural-specific genes increased in the second week with their highest expression in the NEx group. **(C)** TH, Nurr1 and onecut followed nearly the expression pattern of neuronal genes, and their expression increased in the NEx group compared to the other groups. Despite Oct3/4 and Nanog, the expression of Sox-2 significantly enhanced in the NEx group. **(D)** During the second week of differentiation the expression of other specific markers of dopaminergic neurons including Lmx1a and Lmx1b was significantly increased in the NEx group. Foxa2 gene expression was at its highest level in the EEx group. Furthermore, Girk2; a marker preferentially expressed by neurons of the substantia nigra, showed significant enhancement in the NEx group. The experiments were carried out at least in triplicate. Bars represent standard error and different lowercase letters indicate significant differences by LSD test at *P* < 0.05, *n* = 3. For the variables with same letters, the difference is not statistically significant, and for the variables with different letters, the difference is statistically significant. P19, EBs with no treatment (α-MEM + 5% FBS); Ex, EBs treated by brain extract (α-MEM + 5% FBS + 100 μg/ml NRBE); E (empty), EBs transfected with pCMV3-GFPSpark vector; EEx (empty/extract), EBs transfected with pCMV3-GFPSpark vector and treated by extract; N (Nurr), EBs transfected with pCMV3-NR4A2-GFPSpark vector; NEx (Nurr/extract), EBs transfected with pCMV3-NR4A2-GFPSpark vector and treated by extract; W1, W2, W3, week 1, 2, 3, respectively.

In order to investigate the effects of various treatment conditions on the differentiation of P19 EC cells into neuron-like cells with emphasis on dopaminergic neuron-like cells phenotype, the gene expression profile of the cells was quantitatively analyzed by real-time PCR (Figure 4). Overall, the results showed that in the second week of the treatment, the expression levels of stem cell markers reduced significantly. At the same time, there was an enhancement in the expression of neuroepithelial, neural, and dopaminergic specific markers. At this time, the expression of Sox-2 was lowest in the N group, and the lowest level of Oct3/4 gene expression was in N, NEx, EEx, and Ex groups. Although the expression of Nanog decreased in the second week, it was at its lowest level in the NEx group in the third week (Figure 4A). Neuroepithelial (nestin) and neural-specific markers (synaptophysin, BDNF, NGF) and CREB tend to increase in the second week, and their highest expression was in the NEx group (Figure 4B). The expression patterns of dopaminergic specific genes (TH, Nurr1) and onecut were similar to those of neuronal genes, so that they tend to elevate in the second week, and the highest level of their expression was in the NEx group (Figure 4C). The results demonstrated that with a few exceptions, nearly all the genes had a similar pattern of expression. Although stem cell markers showed a reduction pattern in their expression, the expression of Sox-2 increased significantly in the NEx group. Despite nestin, expression of synaptophysin and CREB decreased in the N group, but levels of all three genes increased in the NEx group. Interestingly, BDNF, NGF, TH, Nurr1, and onecut genes were concerts in their expression patterns, so that they had the highest expression level in the NEx group. The expression of other markers of dopaminergic neurons including Lmx1a, Lmx1b, Foxa2, and Girk2 (a marker preferentially expressed by neurons of the substantia nigra) was also evaluated (Figure 4D) in the second week of differentiation. The results confirmed the synergistic effects of Nurr1 overexpression and NRBE on Lmx1a, Lmx1b, and Girk2 level in the NEx group. The expression of Foxa2 was significantly increased in EEx and NEx groups. Since the results of Q-PCR confirmed that there was no significant difference between the two control groups, P19 and E, the subsequent analyses continued with only the E group as control.

### Evaluation of Protein Expression by Immunofluorescence and Flow Cytometry

**Figure 5:**
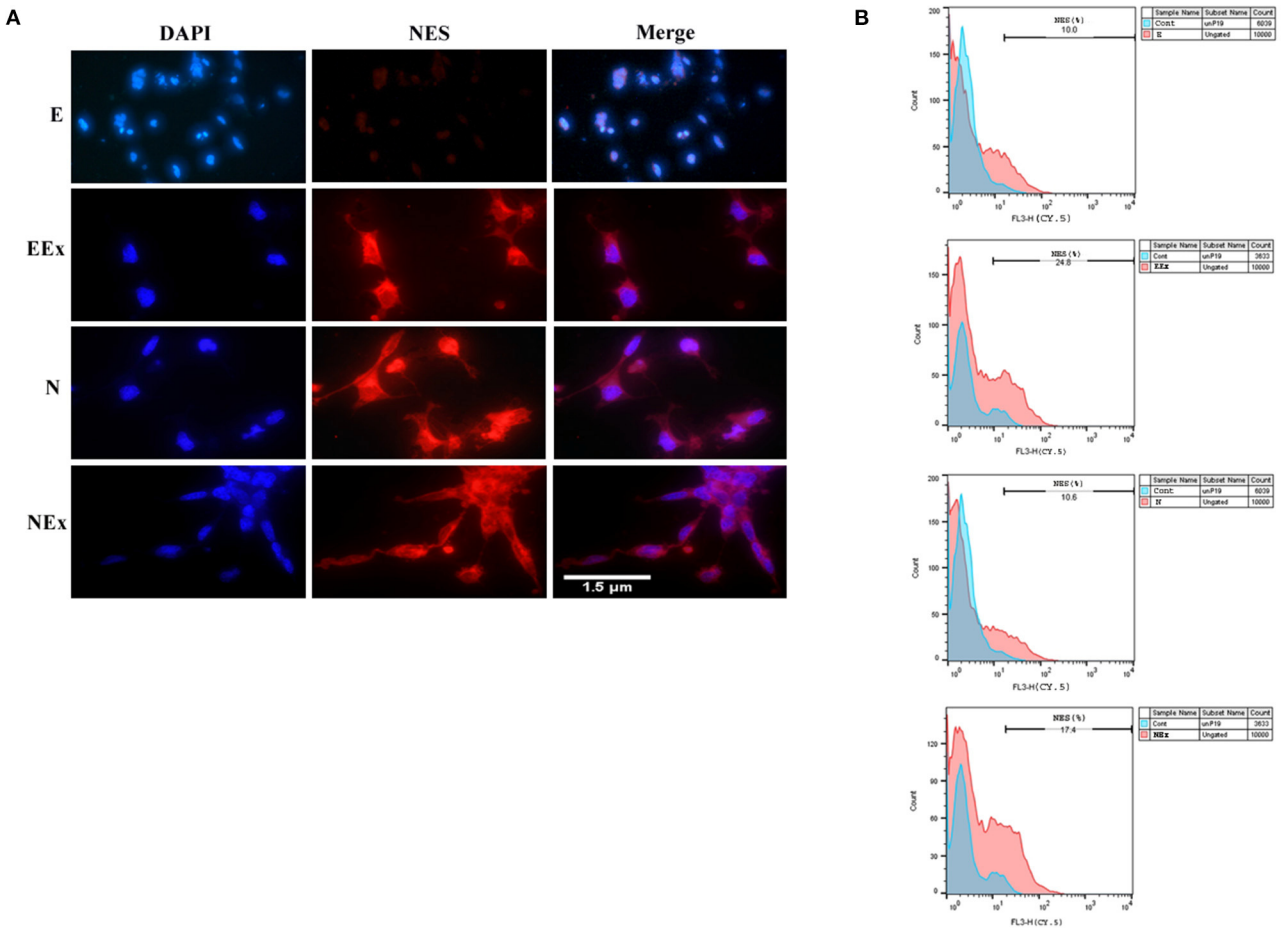
Assessment of neuroepithelial marker, nestin in the second week of the differentiation protocol by: **(A)** immunofluorescence, and **(B)** flow cytometry. The results showed that the cells of all three experimental groups EEx, N, and NEx, were immunoreactive to nestin and the expression of this protein was higher in EEx and NEx groups. The cells in the E group showed no reactivity to nestin. DAPI was applied to counterstain the nuclei. NES, nestin; E (empty), EBs transfected with pCMV3-GFPSpark vector; EEx (empty/extract), EBs transfected with pCMV3-GFPSpark vector and treated by extract; N (Nurr), EBs transfected with pCMV3-NR4A2-GFPSpark vector; NEx (Nurr/extract), EBs transfected with pCMV3-NR4A2-GFPSpark vector and treated by extract.

According to the preliminary results from the morphological studies and the results of gene expression evaluation, further analysis of all experimental groups focused on the second week of the differentiation protocol via various methods, including immunofluorescence and flow cytometry. Immunofluorescence assessments confirmed that the cells treated under different conditions were immunoreactive to neuroepithelial marker nestin (Figure 5A). However, there was no distinct positive response in the E group in the second week of analysis. Furthermore, the expression of nestin protein was quantified by flow cytometry (Figure 5B). The most nestin expression by the differentiated cells was in EEx and N groups (24.8 and 17.4%, respectively). There was no evident expression of nestin in the E and N groups (10 and 10.6%, respectively).

**Figure 6:**
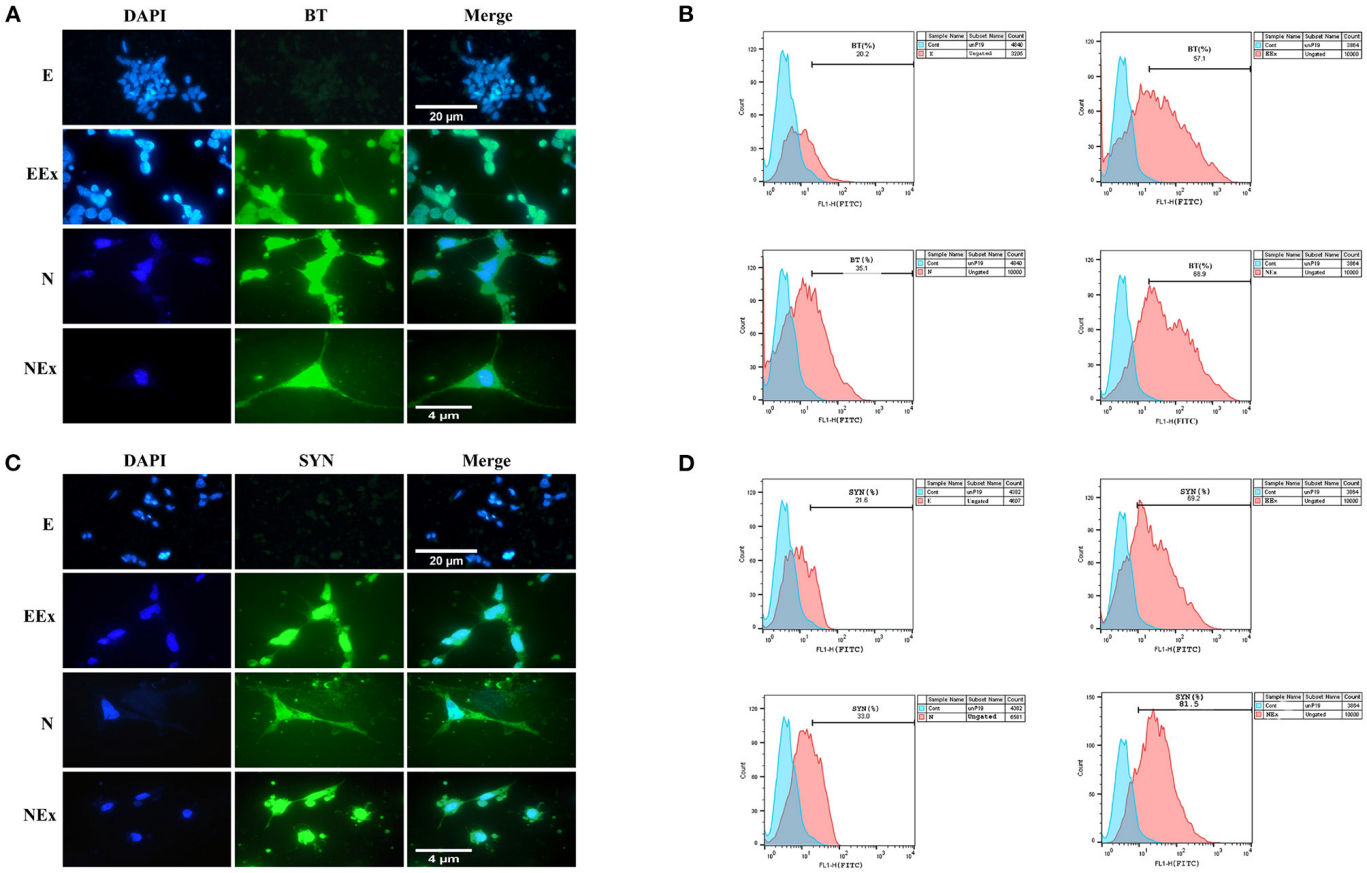
Qualitative and quantitative assessment of neuronal-specific proteins, β-III tubulin, and synaptophysin in the second week of the differentiation protocol. **(A)** The cells of all groups except E were immunoreactive to β-III tubulin. **(B)** Flow cytometry results showed a higher level of β-III tubulin protein in NEx, EEx, and N groups compared to the E group. **(C)** The cells of all groups except E were immunoreactive to synaptophysin. **(D)** Flow cytometry results showed a higher level of synaptophysin protein in NEx, EEx, and N groups compared to the E group. DAPI was applied to counterstain the nuclei. BT, β-III tubulin; SYN, synaptophysin; E (empty), EBs transfected with pCMV3-GFPSpark vector; EEx (empty/extract), EBs transfected with pCMV3-GFPSpark vector and treated by extract; N (Nurr), EBs transfected with pCMV3-NR4A2-GFPSpark vector; NEx (Nurr/extract), EBs transfected with pCMV3-NR4A2-GFPSpark vector and treated by extract.

To evaluate the expression of neuronal-specific proteins, the cells of all the groups were qualitatively and quantitatively analyzed by immunofluorescence and flow cytometry (Figure 6). The expression of pan-neuronal markers, β-III tubulin, and synaptophysin was confirmed in all groups except E (Figures 6A,C). The results from flow cytometry analysis showed that in EEx and NEx groups, the expression of β-III tubulin (57.1 and 68.9%, respectively) and synaptophysin (69.2 and 81.5%, respectively) was at the highest level. While, the expression of β-III tubulin (20.2 and 35.1%, respectively) and synaptophysin (21.8 and 33.0%, respectively) was lower in E and N groups (Figures 6B,D) in comparison with the other groups.

**Figure 7:**
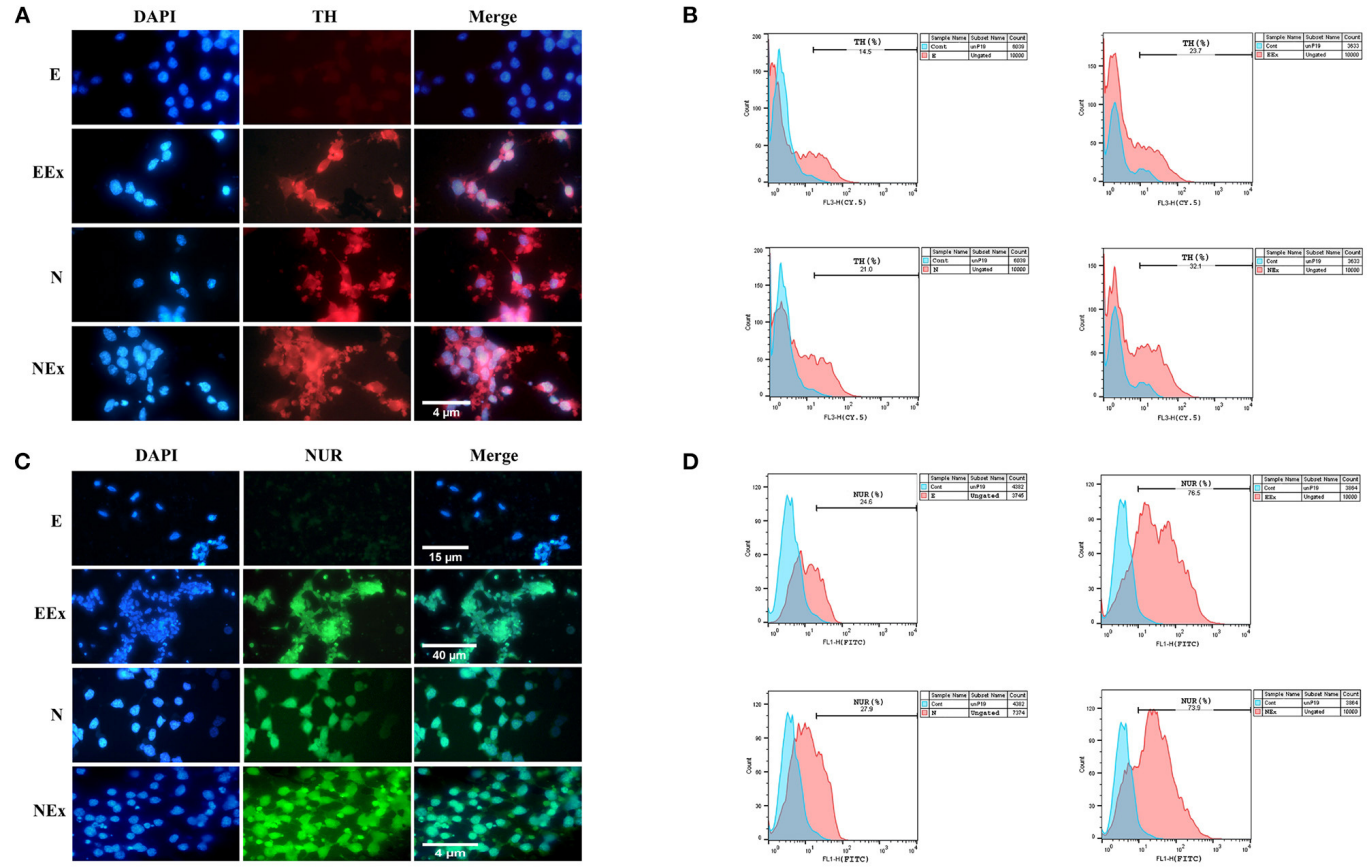
Qualitative and quantitative assessment of dopaminergic specific proteins, tyrosine hydroxylase, and Nurr1 in the second week of the differentiation protocol. **(A)** The cells of all groups except E were immunoreactive to tyrosine hydroxylase. **(B)** Flow cytometry results showed a higher level of tyrosine hydroxylase protein in NEx, EEx, and N groups compared to the E group. **(C)** The cells of all groups except E were immunoreactive to Nurr1. **(D)** Flow cytometry results showed a higher level of Nurr1 protein in NEx, EEx, and N groups compared to the E group. DAPI was applied to counterstain the nuclei. TH, tyrosine hydroxylase; NUR, Nurr1; E (empty), EBs transfected with pCMV3-GFPSpark vector; EEx (empty/extract), EBs transfected with pCMV3-GFPSpark vector and treated by extract; N (Nurr), EBs transfected with pCMV3-NR4A2-GFPSpark vector; NEx (Nurr/extract), EBs transfected with pCMV3-NR4A2-GFPSpark vector and treated by extract.

Furthermore, in the second week of the differentiation protocol, the expression of dopaminergic specific proteins, tyrosine hydroxylase, and Nurr1 in the cells of all groups was analyzed by immunofluorescence and flow cytometry (Figure 7). The expression of these specific markers was confirmed in all groups except E (Figures 7A,C). Flow cytometry analysis showed that in EEx and NEx groups, the expression of TH (23.7 and 32.1%, respectively) and Nurr1 (76.5 and 73.9%, respectively) was at the highest level. While, the expression of TH (14.5 and 21.0%, respectively) and Nurr1 (24.6 and 27.9%, respectively) was lower in E and N groups (Figures 7B,D) compared to other groups.

**Figure 8:**
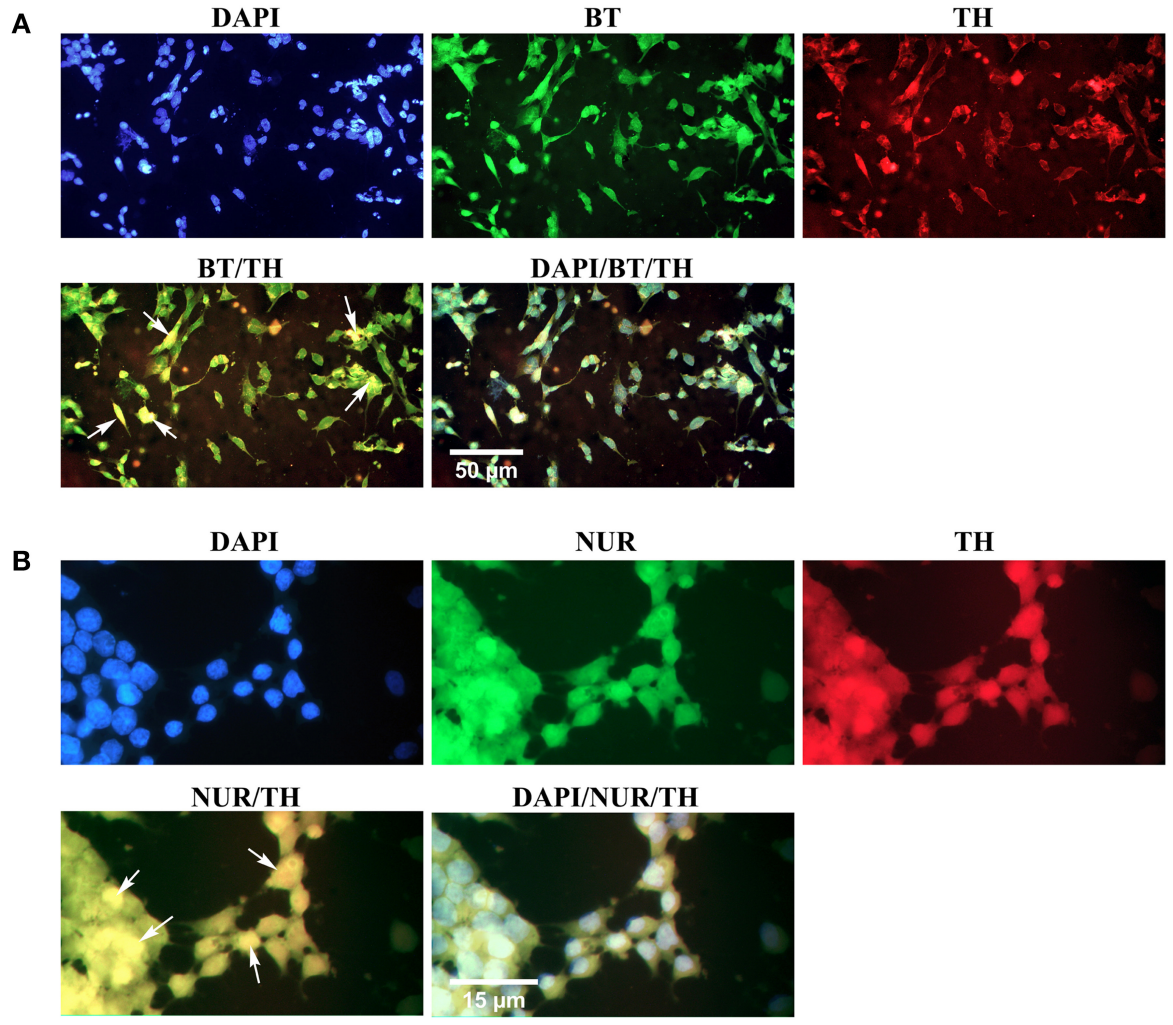
Simultaneous expression of dopaminergic specific proteins in neuron-like cells in the second week of the differentiation protocol. **(A)** Double immunofluorescence staining with antibodies against β-III tubulin (BT, green) and tyrosine hydroxylase (TH, red) confirmed co-expression of a pan-neuronal marker and a dopaminergic specific protein in the differentiated cells (arrows, yellow). **(B)** Double immunofluorescence staining with antibodies against Nurr1 (NUR, green) and TH confirmed co-expression of these dopaminergic specific proteins in the differentiated cells (arrows, yellow). DAPI was applied to counterstain the nuclei. BT, β-III tubulin; TH, tyrosine hydroxylase; NUR, Nurr1.

Simultaneous expression of specific proteins in the differentiated cells was evaluated through double immunofluorescence (Figure 8). The results confirmed that the majority of β-III tubulin-expressing neural cells were also immunoreactive to tyrosine hydroxylase, which is a valid marker for the dopaminergic phenotype (Figure 8A). In addition, some differentiated cells expressed two dopaminergic specific proteins, Nurr1, and tyrosine hydroxylase, simultaneously (Figure 8B). Yellow-stained cytoplasm in the representative fluorescence micrographs (arrows) illustrated co-expression of two proteins in the same cells.

## Discussion

In the present study, we have shown that combined molecular cloning and tissue-specific induction strategies could efficiently enhance the neural differentiation of stem cells with dopaminergic neuron-like phenotypes. In order to differentiation induction, P19 EC stem cells were stably transfected by a plasmid containing a dopaminergic key transcription factor, Nurr1, and then exposed to a medium supplied by a developing rat brain extract NRBE. The results demonstrated that although Nurr1 overexpression or NREB individually induced dopaminergic neuron-like differentiation, a combination strategy had more significant effects. Results from morphological, cellular, and molecular studies confirmed DA-like cell characterizations in the differentiated cells.

Degeneration of DA neurons which are critical for controlling key functions of the brain, is associated with neurological diseases such as PD. Using drugs such as monoamine oxidase-B inhibitors, L-DOPA, and dopamine receptor agonists to treat this disease is not always possible for a long time due to their side effects. An alternative method for PD treatment is the replacement of degenerated cells by DA cells produced from stem cells (Politis and Lindvall, 2012). So far, various protocols have been reported to generate such cells for different aims including, studies on mechanisms of DA neuron development, PD pathogenesis, pharmaceutical approaches, and cell therapy.

Applying extrinsic growth factors, i.e., small molecules and soluble reagents in combination with molecular cloning strategies during *in vitro* differentiation of stem cells, provides an important tool to generate specific cells for PD therapy. Both extrinsic and intrinsic factors impact the determination of cell fate during development. Soluble extrinsic factors secreted by developing tissues in concert with intrinsic transcription factors provide a unique microenvironment for cell differentiation (Kim, 2011). For instance, differentiation of DA neurons requires the concerted action of extrinsic (Shh, FGF8, and Wnt1) and intrinsic (Foxa1/2, En1/2, Lmx1a/b, Pitx3, and Nurr1) signals (Joksimovic et al., 2009; Chakrabarty et al., 2012). To induce a certain differentiation fate in stem cells, essential intrinsic factors can be introduced into the cells through cloning strategies, and or soluble extrinsic factors can be added to the culture media as supplements. Kawasaki et al. (2000) showed that SDIA (stromal cell-derived inducing activity) was able to mediate stem cell differentiation into tyrosine hydroxylase-positive neurons without the use of either retinoic acid (RA) or EBs (Kawasaki et al., 2000). Furthermore, it reported that exogenous expression of Pitx3 could raise the gene and protein level of two neurotrophic factors, BDNF and GDNF (glial-derived neurotrophic factor) (Peng et al., 2007). In the previous study, we showed that neonatal whole brain extract could induce DA phenotype with high expression of TH gene in P19 cells. In addition, LC-MS/MS analysis confirmed the presence of factors related to neuronal differentiation in NRBE content (Momendoust et al., 2019). The existence of cytokines, growth factors, and some other peptides in the developing and adult rat brain tissue has been proved previously (Pettmann et al., 1986; Bartlett et al., 1991; Lindholm et al., 1992; Lee et al., 1993; Mogi et al., 1994). This study aimed to address whether or not the overexpression of Nurr1 with a combination of tissue-specific microenvironments could efficiently induce DA neuronal differentiation of P19 cells. Therefore, Nurr1 as an intrinsic factor, and NREB as a source of soluble extrinsic factors applied to generate the desired neuronal phenotype *in vitro*.

Many studies demonstrated that orphan nuclear receptor Nurr1 plays a critical role in development, differentiation, and functional maintenance of midbrain DA neurons. It has been reported that Nurr1 gene overexpression in conjunction with factors derived from type 1 astrocytes of midbrain induced differentiation of a mouse neural stem cell line into DA neurons (Wagner et al., 1999). Furthermore, overexpression of paired like homeodomain 3 (Pitx3) enhanced the expression of BDNF and GDNF (Peng et al., 2007) and also induced TH expression (Messmer et al., 2007). A combination of Nurr1 and Pitx3 overexpression could differentiate mouse induced pluripotent stem cell (iPSC) into functional DA neurons (Salemi et al., 2016). The results of the present study revealed that the expression of neuronal and dopaminergic genes is upregulated in Nurr1-overexpressing cells (N group) compared to controls (P19 and E groups). However, this upregulation was significantly higher when P19 cells were exposed to NRBE (Ex group). The expression of those genes was even more remarkable in the NEx group (Nurr1-overexpressing cells treated by the extract) compared to all control and experimental groups. Indeed, using combined strategy had synergistic effects on DA differentiation of stem cells. Interestingly, the expression pattern of these genes was highly coordinated. The results confirmed the efficient expression of all DA neuron markers in the NEx group in the second week of the differentiation protocol. Previously, it has been reported that brain extracts have several activities concerning central (CNS) and peripheral (PNS) nervous system neurons. Combinations of NGF and rat brain extract have synergistic effects on the survival of chick embryo DRG (dorsal root ganglion) cells (Sensenbrenner et al., 1978). Initial studies have shown that rat brain extract promoted neurite outgrowth from explants of fetal rat retina in culture (Turner et al., 1982). Brain extract contains many soluble factors, including glial growth factor (GGF), N-Acetylaspartate (NAA), *N*-acetyltransferase (NAT), C-type natriuretic peptide (CNP), and protein kinase C (PKC). GGF stimulates the proliferation of purified rat Schwann cells and astrocytes in tissue culture (Brain extract). NAA was initially identified in brain extracts in 1956 (Tallan et al., 1956). In addition, NAT and its corresponding gene, *Nat8l*, are expressed in the brain (Truckenmiller et al., 1985; Ariyannur et al., 2010; Wiame et al., 2010). PKC, first identified in bovine brain extracts (BBE), can regulate cell proliferation, differentiation, and motility. BBE stimulates the growth and expression of cell-specific genes in specialized cell types, including endothelial cells, smooth muscle cells, keratinocytes, melanocytes, hybridomas, primary neuronal cells. It also promotes the differentiation of pluripotent stem cells (Hoshijima et al., 2004).

These findings are in accordance with previous studies demonstrating that tissue-specific microenvironment could induce tissue-specific gene expression in stem cells (Bentz et al., 2010; Ebrahimie et al., 2014; Narayanan et al., 2014; Mansouri et al., 2016; Mehrfarjam et al., 2016). Our results showed that although overexpression of Nurr1 could induce neuronal and dopaminergic markers in stem cells, exposure of the cells to brain extract had synergistic effects on this process. While Nurr1 expression is critical for DA neuronal development, solely its expression is not sufficient to stimulate this phenotype. It has demonstrated that to reach full mature dopaminergic neurons from *in vitro* differentiation of stem cells, additional factors are required (Kim et al., 2003). Therefore, it suggested that the presence of possible factors/proteins in NRBE is involved in regulating Nurr1 gene function. Soluble factors such as BDNF, GDNF, and TGFα (transforming growth factor α) all play an essential role in the differentiation and survival of dopaminergic neurons and also maintenance of their normal functions. Soluble extract derived from striatum could enhance *in vitro* development of mesencephalic DA neurons (Tomozawa and Appel, 1986). The production of DA neurons from stem cells has been stimulated by fetal midbrain astrocytes (Roy et al., 2006). GDNF, neurturin, TGF-ß, and dibutyryl-cAMP as soluble factors promoted the differentiation, maturation, and survival of TH-positive neurons derived from ES cells (Rolletschek et al., 2001). Furthermore, interleukin-1 could significantly increase the number of TH-expressing cells isolated from the midbrain of rat embryos (Ling et al., 1998).

As mentioned above, the expression of neuronal and dopaminergic markers significantly increased in the second week of differentiation. It is possible that BDNF, which is involved in the neuroprotective pathways of dopaminergic neurons, is highly associated with Nurr1. Among the genes that CREB controls are BDNF, TH, and Nurr1. Interaction of CREB with its transcriptional binding site of Nurr1 promoter induces the upregulation of this gene (Lonze and Ginty, 2002; Lei et al., 2011; Dong et al., 2016; Jang et al., 2021). Expression analysis of Onecut1 (Oc1, also called Hnf6) transcription factor during our differentiation system showed the coordinated expression pattern of this gene with other DA-specific genes. The role of Oc transcription factors in dopaminergic development is not yet clear exactly. Matched Oc and Lmx1a expression profiles suggested the involvement of Oc in early DA development (Chakrabarty et al., 2012). The exact functions of the Oc family and their interaction with other critical factors in the development of DA are not yet fully understood and require further investigation.

It is interesting to note that concomitant with an enhancement of neuronal and dopaminergic markers, the expression of stem cell markers was down-regulated. However, the expression of Sox-2 in the NEx group increased exceptionally compared to the other groups. The core transcription factors for regulating pluripotency are Oct3/4, Sox-2, and Nanog (Chen et al., 2008). Furthermore, Sox-2 plays an essential role in the neural differentiation of stem cells and maintaining the properties of neural progenitor cells. Sox-2 can control pluripotency and neural differentiation of human pluripotent stem cells (Khoo et al., 2013; Zhang and Cui, 2014). Downregulation of Sox-2 in the differentiated cells derived from stem cells has been well-established previously (Ebrahimie et al., 2014; Mehrfarjam et al., 2016; Momendoust et al., 2019). IHowever, it is reported that depending on the environmental input, Sox-2 could function as a pluripotency and, or neural differentiation factor (Zhang et al., 2019). Therefore, the high expression of Sox-2 here might reflect its possible role in neural differentiation rather than stem cell proliferation.

## Conclusion

To the best of our knowledge, this work is the first attempt to evaluate the combined effects of Nurr1 gene overexpression with a natural tissue-specific medium on dopaminergic differentiation of P19 EC stem cells. To this end, NREB as a source of extrinsic factors, and Nurr1 as an intrinsic factor, was applied for differentiation induction. Overall, the results of the present study demonstrated the synergistic effects of Nurr1 gene overexpression and possible soluble factors that existed in NRBE on differentiation induction of P19 stem cells into neuronal cells with a dopaminergic neuron-like phenotype. Although therapies based on molecular targets for PD sound promising, the feasibility and safety of Nurr1 gene therapy still need further improvement.

## Data Availability Statement

The original contributions presented in the study are included in the article/Supplementary Material, further inquiries can be directed to the corresponding author/s.

## Ethics Statement

The animal study was reviewed and approved by Bioethics Committee of the University of Isfahan, based on the National Specific Ethical Guidelines for Biomedical Research issued by the Ministry of Health and Medicinal Education (MOHME) of Iran in 2005.

## Author Contributions

FE conceived and designed the experiments, analyzed the data, and wrote the paper. RB, MK, FE, and FD performed the experiments. All authors contributed to the article and approved the submitted version.

## Funding

This research was performed at the Department of Plant and Animal Biology, Faculty of Biological Science and Technology, University of Isfahan, Isfahan, Iran. It was supported by the research fund of the University of Isfahan (grant number 96/29696). The funders had no role in the design of the study, data collection and analysis, preparation of the manuscript, and decision to publish.

## Conflict of Interest

The authors declare that the research was conducted in the absence of any commercial or financial relationships that could be construed as a potential conflict of interest.

## Publisher's Note

All claims expressed in this article are solely those of the authors and do not necessarily represent those of their affiliated organizations, or those of the publisher, the editors and the reviewers. Any product that may be evaluated in this article, or claim that may be made by its manufacturer, is not guaranteed or endorsed by the publisher.

## References

[B1] Ariyannur P. S. Moffett J. R. Manickam P. Pattabiraman N. Arun P. Nitta A.. (2010). Methamphetamine-induced neuronal protein NAT8L is the NAA biosynthetic enzyme: implications for specialized acetyl coenzyme A metabolism in the CNS. Brain Res. 1335, 1–13. 10.1016/j.brainres.2010.04.008 20385109

[B2] Azizi F. Jalil H. Nasiri Z. Moshtaghian J. Esmaeili F. Doostmohammadi A.. (2018). The combined effects of three dimensional cell culture and natural tissue extract on neural differentiation of P19 embryonal carcinoma stem cells. J. Tissue Eng. Regen. Med. 12, 1909–1924. 10.1002/term.2712 29905008

[B3] Bartlett W. Li X.-S. Williams M. Benkovic S. (1991). Localization of insulin-like growth factor-1 mRNA in murine central nervous system during postnatal development. Dev. Biol. 147, 239–250. 10.1016/S0012-1606(05)80021-1 1879610

[B4] Bartus R. T. Baumann T. L. Siffert J. Herzog C. D. Alterman R. Boulis N.. (2013). Safety/feasibility of targeting the substantia nigra with AAV2-neurturin in Parkinson patients. Neurology 80, 1698–1701. 10.1212/WNL.0b013e3182904faa 23576625 PMC3716474

[B5] Bentz K. Molcanyi M. Schneider A. Riess P. Maegele M. Bosche B.. (2010). Extract derived from rat brains in the acute phase following traumatic brain injury impairs survival of undifferentiated stem cells and induces rapid differentiation of surviving cells. Cell. Physiol. Biochem. 26, 821–830. 10.1159/000323991 21220913

[B6] Chakrabarty K. Von Oerthel L. Hellemons A. Clotman F. Espana A. Groot Koerkamp M.. (2012). Genome wide expression profiling of the mesodiencephalic region identifies novel factors involved in early and late dopaminergic development. Biol. Open 1, 693–704. 10.1242/bio.20121230 23213462 PMC3507229

[B7] Chen C. Okayama H. (1987). High-efficiency transformation of mammalian cells by plasmid DNA. Mol. Cell. Biol. 7, 2745–2752. 10.1128/MCB.7.8.2745 3670292 PMC367891

[B8] Chen X. Xu H. Yuan P. Fang F. Huss M. Vega V. B.. (2008). Integration of external signaling pathways with the core transcriptional network in embryonic stem cells. Cell 133, 1106–1117. 10.1016/j.cell.2008.04.043 18555785

[B9] Decressac M. Kadkhodaei B. Mattsson B. Laguna A. Perlmann T. Björklund A. (2012). α-Synuclein–induced down-regulation of Nurr1 disrupts GDNF signaling in nigral dopamine neurons. Sci. Transl. Med. 4, 163ra156. 10.1126/scitranslmed.3004676 23220632

[B10] Dong J. Li S. Mo J. L. Cai H. B. Le W. D. (2016). Nurr1-based therapies for Parkinson's disease. CNS Neurosci. Ther. 22, 351–359. 10.1111/cns.12536 27012974 PMC4833611

[B11] Ebrahimie M. Esmaeili F. Cheraghi S. Houshmand F. Shabani L. Ebrahimie E. (2014). Efficient and simple production of insulin-producing cells from embryonal carcinoma stem cells using mouse neonate pancreas extract, as a natural inducer. PLoS ONE 9:e90885. 10.1371/journal.pone.0090885 24614166 PMC3948699

[B12] Fraichard A. Chassande O. Bilbaut G. Dehay C. Savatier P. Samarut J. (1995). *In vitro* differentiation of embryonic stem cells into glial cells and functional neurons. J. Cell Sci. 108, 3181–3188. 10.1242/jcs.108.10.3181 7593279

[B13] Han C. Song L. Liu Y. Zou W. Jiang C. Liu J. (2014). Rat cortex and hippocampus-derived soluble factors for the induction of adipose-derived mesenchymal stem cells into neuron-like cells. Cell Biol. Int. 38, 768–776. 10.1002/cbin.10256 24500988

[B14] Hermanson E. Joseph B. Castro D. Lindqvist E. Aarnisalo P. Wallén Å.. (2003). Nurr1 regulates dopamine synthesis and storage in MN9D dopamine cells. Exp. Cell Res. 288, 324–334. 10.1016/S0014-4827(03)00216-7 12915123

[B15] Hoshijima M. Minamisawa S. Yasukawa H. Chien K. R. (2004). Molecular pathways for cardiac hypertrophy and heart failure progression. Mol. Basis Cardiovasc. Dis. 15, 273–292. 10.1016/B978-0-7216-9428-3.50020-231849158

[B16] Jang Y. Kim W. Leblanc P. Kim C.-H. Kim K.-S. (2021). Potent synthetic and endogenous ligands for the adopted orphan nuclear receptor Nurr1. Exp. Mol. Med. 53, 19–29. 10.1038/s12276-021-00555-5 33479411 PMC8080818

[B17] Joksimovic M. Yun B. A. Kittappa R. Anderegg A. M. Chang W. W. Taketo M. M.. (2009). Wnt antagonism of Shh facilitates midbrain floor plate neurogenesis. Nat. Neurosci. 12, 125–131. 10.1038/nn.2243 19122665

[B18] Kawasaki H. Mizuseki K. Nishikawa S. Kaneko S. Kuwana Y. Nakanishi S.. (2000). Induction of midbrain dopaminergic neurons from ES cells by stromal cell–derived inducing activity. Neuron 28, 31–40. 10.1016/S0896-6273(00)00083-0 11086981

[B19] Khoo T. S. Hamidah Hussin N. Then S.-M. Jamal R. (2013). Autogenic feeder free system from differentiated mesenchymal progenitor cells, maintains pluripotency of the MEL-1 human embryonic stem cells. Differentiation 85, 110–118. 10.1016/j.diff.2013.01.004 23722082

[B20] Kim H.-J. (2011). Stem cell potential in Parkinson's disease and molecular factors for the generation of dopamine neurons. Biochim. Biophys. Acta Mol. Basis Dis. 1812, 1–11. 10.1016/j.bbadis.2010.08.006 20713152

[B21] Kim H.-J. Sugimori M. Nakafuku M. Svendsen C. N. (2007). Control of neurogenesis and tyrosine hydroxylase expression in neural progenitor cells through bHLH proteins and Nurr1. Exp. Neurol. 203, 394–405. 10.1016/j.expneurol.2006.08.029 17034791

[B22] Kim J. Y. Koh H. C. Lee J. Y. Chang M. Y. Kim Y. C. Chung H. Y.. (2003). Dopaminergic neuronal differentiation from rat embryonic neural precursors by Nurr1 overexpression. J. Neurochem. 85, 1443–1454. 10.1046/j.1471-4159.2003.01780.x 12787064

[B23] Lee W.-H. Michels K. Bondy C. (1993). Localization of insulin-like growth factor binding protein-2 messenger RNA during postnatal brain development: correlation with insulin-like growth factors I and II. Neuroscience 53, 251–265. 10.1016/0306-4522(93)90303-W 7682300

[B24] Lei Z. Jiang Y. Li T. Zhu J. Zeng S. (2011). Signaling of glial cell line-derived neurotrophic factor and its receptor GFRα1 induce Nurr1 and Pitx3 to promote survival of grafted midbrain-derived neural stem cells in a rat model of Parkinson disease. J. Neuropathol. Exp. Neurol. 70, 736–747. 10.1097/NEN.0b013e31822830e5 21865882

[B25] Lindholm D. Castren E. Kiefer R. Zafra F. Thoenen H. (1992). Transforming growth factor-beta 1 in the rat brain: increase after injury and inhibition of astrocyte proliferation. J. Cell Biol. 117, 395–400. 10.1083/jcb.117.2.395 1560032 PMC2289420

[B26] Ling Z. D. Potter E. D. Lipton J. W. Carvey P. M. (1998). Differentiation of mesencephalic progenitor cells into dopaminergic neurons by cytokines. Exp. Neurol. 149, 411–423. 10.1006/exnr.1998.6715 9500954

[B27] Lonze B. E. Ginty D. D. (2002). Function and regulation of CREB family transcription factors in the nervous system. Neuron 35, 605–623. 10.1016/S0896-6273(02)00828-0 12194863

[B28] Mansouri A. Esmaeili F. Nejatpour A. Houshmand F. Shabani L. Ebrahimie E. (2016). Differentiation of P19 embryonal carcinoma stem cells into insulin-producing cells promoted by pancreas-conditioned medium. J. Tissue Eng. Regen. Med. 8, 600–612. 10.1002/term.1927 25044225

[B29] Mehrfarjam Z. Esmaeili F. Shabani L. Ebrahimie E. (2016). Induction of pancreatic β cell gene expression in mesenchymal stem cells. Cell Biol. Int. 40, 486–500. 10.1002/cbin.10567 26634639

[B30] Messmer K. Remington M. P. Skidmore F. Fishman P. S. (2007). Induction of tyrosine hydroxylase expression by the transcription factor Pitx3. Int. J. Dev. Neurosci. 25, 29–37. 10.1016/j.ijdevneu.2006.11.003 17184956

[B31] Mogi M. Harada M. Kondo T. Riederer P. Inagaki H. Minami M.. (1994). Interleukin-1β, interleukin-6, epidermal growth factor and transforming growth factor-α are elevated in the brain from parkinsonian patients. Neurosci. Lett. 180, 147–150. 10.1016/0304-3940(94)90508-8 7700568

[B32] Momendoust N. Moshtaghian J. Esmaeili F. Dehghanian F. Dumit V. (2019). Induction of Tyrosine Hydroxylase Gene Expression in Embryonal Carcinoma Stem Cells Using a Natural Tissue-Specific Inducer. Dev. Neurobiol. 79, 559–577. 10.1002/dneu.22703 31177638

[B33] Murphy E. P. Conneely O. M. (1997). Neuroendocrine regulation of the hypothalamic pituitary adrenal axis by the nurr1/nur77 subfamily of nuclear receptors. Mol. Endocrinol. 11, 39–47. 10.1210/mend.11.1.9874 8994186

[B34] Narayanan K. Lim V. Y. Shen J. Tan Z. W. Rajendran D. Luo S.-C.. (2014). Extracellular matrix-mediated differentiation of human embryonic stem cells: differentiation to insulin-secreting beta cells. Tissue Eng. Part A 20, 424–433. 10.1089/ten.tea.2013.0257 24020641

[B35] Peng C. Fan S. Li X. Fan X. Ming M. Sun Z.. (2007). Overexpression of pitx3 upregulates expression of BDNF and GDNF in SH-SY5Y cells and primary ventral mesencephalic cultures. FEBS Lett. 581, 1357–1361. 10.1016/j.febslet.2007.02.054 17350004

[B36] Pettmann B. Labourdette G. Weibel M. Sensenbrenner M. (1986). The brain fibroblast growth factor (FGF) is localized in neurons. Neurosci. Lett. 68, 175–180. 10.1016/0304-3940(86)90137-0 3748449

[B37] Politis M. Lindvall O. (2012). Clinical application of stem cell therapy in Parkinson's disease. BMC Med. 10, 1–7. 10.1186/1741-7015-10-1 22216957 PMC3261810

[B38] Rivera F. J. Couillard-Despres S. Pedre X. Ploetz S. Caioni M. Lois C.. (2006). Mesenchymal stem cells instruct oligodendrogenic fate decision on adult neural stem cells. Stem Cells 24, 2209–2219. 10.1634/stemcells.2005-0614 16763198

[B39] Rolletschek A. Chang H. Guan K. Czyz J. Meyer M. Wobus A. M. (2001). Differentiation of embryonic stem cell-derived dopaminergic neurons is enhanced by survival-promoting factors. Mech. Dev. 105, 93–104. 10.1016/S0925-4773(01)00385-9 11429285

[B40] Roy N. S. Cleren C. Singh S. K. Yang L. Beal M. F. Goldman S. A. (2006). Functional engraftment of human ES cell-derived dopaminergic neurons enriched by coculture with telomerase-immortalized midbrain astrocytes. Nat. Med. 12, 1259–1268. 10.1038/nm1495 17057709

[B41] Salemi S. Baktash P. Rajaei B. Noori M. Amini H. Shamsara M.. (2016). Efficient generation of dopaminergic-like neurons by overexpression of Nurr1 and Pitx3 in mouse induced Pluripotent Stem Cells. Neurosci. Lett. 626, 126–134. 10.1016/j.neulet.2016.05.032 27208834

[B42] Sensenbrenner M. Maderspach K. Latzkovits L. Jaros G. (1978). Neuronal cells from chick embryo cerebral hemispheres cultivated on polylysine-coated surfaces. Dev. Neurosci. 1, 90–101. 10.1159/000112560 755685

[B43] Tallan H. H. Moore S. Stein W. H. (1956). N-Acetyl-L-aspartic acid in brain. J. Biol. Chem. 219, 257–264. 10.1016/S0021-9258(18)65789-8 13295277

[B44] Tetradis S. Bezouglaia O. Tsingotjidou A. (2001). Parathyroid hormone induces expression of the nuclear orphan receptor Nurr1 in bone cells. Endocrinology 142, 663–670. 10.1210/endo.142.2.7926 11159837

[B45] Tomozawa Y. Appel S. H. (1986). Soluble striatal extracts enhance development of mesencephalic dopaminergic neurons *in vitro*. Brain Res. 399, 111–124. 10.1016/0006-8993(86)90605-0 3801914

[B46] Truckenmiller M. Namboodiri M. Brownstein M. Neale J. (1985). N-Acetylation of l-aspartate in the nervous system: differential distribution of a specific enzyme. J. Neurochem. 45, 1658–1662. 10.1111/j.1471-4159.1985.tb07240.x 4045470

[B47] Turner J. E. Barde Y.-A. Schwab M. E. Thoenen H. (1982). Extract from brain stimulates neurite outgrowth from fetal rat retinal explants. Dev. Brain Res. 6, 77–83. 10.1016/0165-3806(82)90176-6 7159845

[B48] Wagner J. Akerud P. Castro D. S. Holm P. C. Canals J. M. Snyder E. Y.. (1999). Induction of a midbrain dopaminergic phenotype in Nurr1-overexpressing neural stem cells by type 1 astrocytes. Nat. Biotechnol. 17, 653–659. 10.1038/10862 10404157

[B49] Wiame E. Tyteca D. Pierrot N. Collard F. Amyere M. Noel G.. (2010). Molecular identification of aspartate N-acetyltransferase and its mutation in hypoacetylaspartia. Biochem. J. 425, 127–139. 10.1042/BJ20091024 19807691

[B50] Yuan J. Lei Z.-n. Wang X. Deng Y.-J. Chen D.-B. (2015). Interaction between Oc-1 and Lmx1a promotes ventral midbrain dopamine neural stem cells differentiation into dopamine neurons. Brain Res. 1608, 40–50. 10.1016/j.brainres.2015.02.046 25747864

[B51] Zetterström R. H. Solomin L. Jansson L. Hoffer B. J. Olson L. Perlmann T. (1997). Dopamine neuron agenesis in Nurr1-deficient mice. Science 276, 248–250. 10.1126/science.276.5310.248 9092472

[B52] Zhang S. Bell E. Zhi H. Brown S. Imran S. A. Azuara V.. (2019). OCT4 and PAX6 determine the dual function of SOX2 in human ESCs as a key pluripotent or neural factor. Stem Cell Res. Ther. 10:122. 10.1186/s13287-019-1228-7 30999923 PMC6471829

[B53] Zhang S. Cui W. (2014). Sox2, a key factor in the regulation of pluripotency and neural differentiation. World J. Stem Cells 6, 305–311. 10.4252/wjsc.v6.i3.305 25126380 PMC4131272

