# Supplementary figures and images for: Synergistic Effects of Combined Nurr1 Overexpression and Natural Inducers on the More Efficient Production of Dopaminergic Neuron-Like Cells From Stem Cells
Source: Front Cell Neurosci. 2022 Jan 11;15:803272. doi: 10.3389/fncel.2021.803272 (PMC8787052; doi:10.3389/fncel.2021.803272)

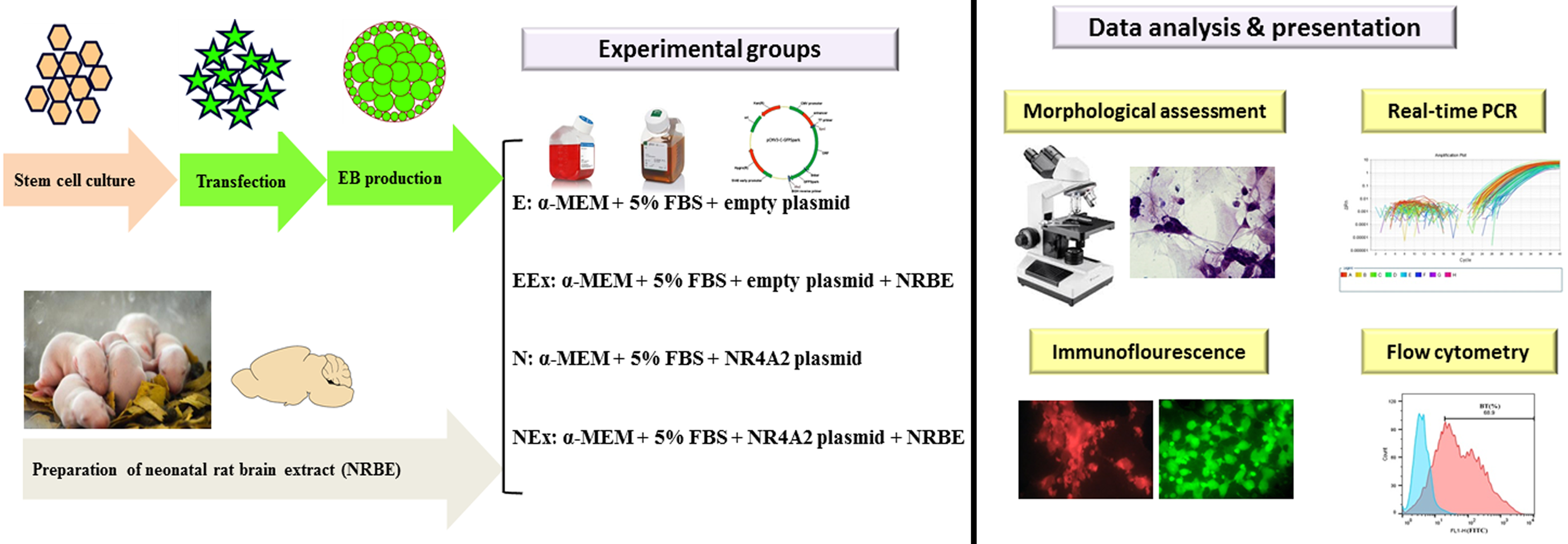

Supplement: Supplementary file 1 [file Image_1.TIF]
